# Perception of Cervical Cancer Patients on their Financial Challenges in Western Kenya

**DOI:** 10.1186/s12913-018-3073-2

**Published:** 2018-04-10

**Authors:** Jane A. Owenga, Erick Otieno Nyambedha

**Affiliations:** 1 Jaramogi Oginga Odinga University of Science and Technology, P.O Box.210-40601, Bondo, Kenya; 2 Department of Sociology and Anthropology, Maseno University, Private Bag, Maseno, Kenya

**Keywords:** Cervical cancer, Financial challenges, Kenya

## Abstract

**Background:**

The number of cervical cancer cases is reported to increase among women of reproductive age in the recent past with patients facing challenges with care and management of the illness. However, little is known about the financial challenges these patients undergo in contexts such as western Kenya. This study assessed financial challenges and sources of financial assistance for cervical cancer patients in western Kenya.

**Methods:**

A cross-sectional study involving 334 cervical cancer patients was conducted in Jaramogi Oginga Odinga Teaching and Referral Hospital (JOOTRH) in Kisumu from September 2014 to February 2015. Structured questionnaire, in-depth interview guide and key informant interview guide were used to collect data. Quantitative data was analyzed using Statistical Package for Social Scientists (SPSS) Version 20 at a statistical significance of *P* ≤ 0.05, descriptive statistics and crosstabulations were performed. For qualitative data, the responses were transcribed verbatim and the content was then analyzed by searching for emerging themes on the financial challenges faced by cervical cancer patients. Qualitative data was presented in textual form with verbatim reports for illustrations.

**Results:**

The key financial challenges from the study were costs of medication 291 (87%), cost of travel 281 (84%) and cost of diagnostic tests 250 (75%). Other costs incurred by the patients were cost of cloths and wigs 91 (27%), and cost of home and child care 80 (24%). Most 304 (91%) of the cervical cancer patients admitted and referred to JOOTRH did not have insurance cover and only 30 (9%) had National Hospital Insurance Fund cover which catered for only bed component of inpatient costs. Results showed that no patient received any assistance from well-wishers. Only a few received assistance from charity organizations 43 (13%), friends 91 (27%) and colleagues 31 (9%). Some patients received some assistance from relatives 32 (10%) and church 32 (10%).

**Conclusion:**

Cervical cancer patients experience several financial challenges yet only few of them had insurance cover which catered for only bed component of inpatient costs. There is a need for the Kenyan health care system to develop mechanisms for provision of financial support for cervical cancer patients.

**Electronic supplementary material:**

The online version of this article (10.1186/s12913-018-3073-2) contains supplementary material, which is available to authorized users.

## Background

Cervical and breast cancers are the leading causes of cancer morbidity and mortality among the female population worldwide [1]. In the year 2011, cancer of the breast and cancer of the cervix were the most prevalent cancers with 1,676,633 and 527,624 new cases of women being diagnosed with the two cancers respectively. Breast cancer is the leading cause of cancer related deaths followed by cervical cancer among female worldwide [1]. In Kenya, current estimates indicate that every year 4802 women are diagnosed with cervical cancer and 2451 die from the disease. Cervical cancer ranks as the first most frequent cancer among women in Kenya [2].

Until recently, little has been known about the costs incurred by cancer patients and their families in the cancer management internationally [3]. Berkman and Sampson [4] argued that there is growing awareness that cancer can have a major financial impact on newly diagnosed patients, those living with the disease and their families, they further report that, it has been claimed that almost all families confronted with a diagnosis of cancer have financial challenges of some kind. Cancer patients are more likely to report financial challenges than persons without cancer [5, 6]. Timmons et al. [7] also noted that patients incurred a wide range of additional cancer related medical and non- medical expenses in Ireland revealing the multidimensional nature of the financial and economic burden cancer imposes on patients and the whole family unit. Cervical cancer patients incur out of pocket expenses as a result of their condition. Longo et al. [8] explained that these out-of-pocket costs are varied and might include: expenses directly due to treatment (doctor’s fees); expenses related to treatment (travel costs, prescription, medication, wigs); or more general expenses that are as a result of having cancer (new clothes due to weight fluctuations, different food or nutritional supplements due to the effects of chemotherapy). In addition, some patients experience a reduction in income due to time taken away from work because of the cancer or its treatment [9]. Those with a low income are particularly vulnerable to the adverse financial and economic effects of incurring out of pocket expenses [10].

Being a cancer patient can lead to catastrophic expenses with several consequences including a reduction in total household earnings due to family adjustments for the disease, having to cut-back on “extras” such as social expenditure or holidays, sale of existing family assets and having to borrow money. Moreover, it can also trigger non-financial consequences, such as financial stress leading to psychological problems [11–14]. In the US more than 2 million cancer survivors did not get one or more needed medical services because of financial challenges, this was mainly seen among the Hispanic and black survivors [15]. Need for financial support to counter the loss of income of both cancer patient and family caregiver has been reported in several countries in Africa [16, 17]. Masika et al. [16], reported financial challenges among cancer patients in Tanzania. However, their study did not explore the actual financial challenges of cervical cancer patients. On the other hand Sepulveda et al. [17], in their study conducted in a couple of African countries (Botswana, Ethiopia, Tanzania, Uganda, and Zimbabwe) among Human Immunodeficiency Virus/ Acquired Immune Deficiency Syndrome (HIV/AIDS) and cancer patients, revealed needs for financial support to counter the loss of income of both patient and family caregiver. Mostert et al. [18] studied contributors to abandonment of childhood cancer treatment in Kenya and established financial burden of cancer treatment and lack of insurance as one of the important predictors to abandonment of treatment. Moreover, long distances to access diagnostic and treatment services, lack of decentralized diagnostic and treatment facilities and a lack of better cancer policy development have been established as factors hindering access to cancer treatment in Kenya [19]. According to Gitonga [20] the total cost of breast cancer treatment at Kenyatta National Hospital (KNH) in Kenya is well above the country’s average wage level and subsidization is required. These studies did not capture financial challenges unique to cervical cancer patients which is currently the leading cause of morbidity and mortality related to cancer among women in Kenya and more so western Kenya. Little literature exists to describe the financial challenges of cervical cancer patients in western Kenya. An important purpose of this study was to determine financial challenges of cervical cancer patients with the aim of using this information to develop evidence based interventions for managing care of cervical cancer patients in Kenya.

The study was conducted at Jaramogi Oginga Odinga Teaching and Referral Hospital (JOOTRH) which is the referral hospital for cancer patients in western part of Kenya. The total in-patient cervical cancer admissions in 2014 and 2015 when the study was conducted were 681 and 735 respectively. The facility did not have special wards for adult cancer patients and therefore the female cancer patients were admitted in gynecological wards. Other infrastructure that were available for cancer included: palliative care/ oncology unit and medical social department which helped in case the patients had social problems. Facilities available for management of cervical cancer were cryotherapy and Loop Electrosurgical Excision Procedure (LEEP) equipment. Radiotherapy machine was available but was not operational. The facility did not have an oncologist, there were three palliative care nurses, a pathologist (mainly conducting biopsies) and gynecologists who staged cervical cancer and conducted surgery where appropriate. The hospital offered the following services for cervical cancer patients: screening, palliative care, chemotherapy, cryotherapy, conization and surgery. For other associated diagnostic and routine laboratory tests the patients would be referred to the neighboring private facilities.

## Methods

### Study setting

The study was conducted at JOOTRH in Kisumu from September 2014 to February 2015. JOOTRH is a referral hospital serving a catchment area with a population of more than 5 million people in more than 10 counties in the western region of Kenya. The hospital serves an area with some of the worst health indicators in the country including high prevalence of HIV infection (15.4%) which is greater than twice that of the national (7.1%) prevalence [21]. JOOTRH is the referral hospital for cancer patients in western part of Kenya. At the time of this study, total in-patient cervical cancer admissions were 681 and 735 in 2014 and 2015 respectively.

### Study design

This was a hospital based cross-sectional descriptive study. It involved collection of both quantitative and qualitative data from cervical cancer patients seeking care at JOOTRH.

### Study participants

The study participants consisted of cervical cancer patients who were over 18 years visiting JOOTRH or referred to JOOTRH for further treatment or palliative care services. The eligible respondents were sourced from palliative care clinics, oncology unit, and obstetric and gynecological unit within JOOTRH.

### Sampling Design

The sampling strategy involved purposive sequential enrolment of patients with histologically proven cervical cancer as they became available at the facility till the required sample size was reached. Medical social workers were also purposively sampled for key informant interview. The healthcare providers in charge of the patients and palliative care specialist helped identify patients based on information in the patient files then referred them to the researcher who confirmed their eligibility and proceeded to seek consent from each of them. This exercise was done every day in the gynecological ward (ward 4) and in room 19/16 where cervical cancer screening, cryotherapy and LEEP was performed. Patients were also selected on Tuesdays in the gynecological outpatient clinics (GOPC) where patients who were diagnosed at early stage were done for surgery. This was done until the desired sample size (334) was achieved. Participants for in-depth interviews were selected by the care providers based on how long they had been symptomatically sick (at least more than one year) and consented to the study. Pilot survey was conducted at the neighboring Kisumu East sub-county hospital to test the study tools and improve their quality and efficiency.

### Research Procedure

A structured questionnaire (see Additional file 1) was administered to the recruited patients by the researcher and research assistants. The researcher liaised with the relevant health care providers at the palliative care unit, obstetric/gynecological wards and on GOPC days which were appropriate for identification of the eligible clients. The researcher and the health care team worked out a programme on how the researcher could access the respondents without putting any strain on the respondents such as by keeping them longer in the facility. The researcher also took contacts of clients who consented to the study but were not able to respond to the questionnaire at that time and made private arrangement with them on when and where it was convenient to administer the questionnaire to them. The participants answered the questionnaires by themselves except for some 21 (6.3%) who needed help and were assisted by the researcher.

Two medical social workers were interviewed (see Additional file 2) by the researcher face to face and their responses were audio recorded and transcribed verbatim. In-depth interview guide (see Additional file 3) was used to collect qualitative data from 12 eligible patients by the researcher. The patients were guided to narrate their story concerning their financial challenges during the disease trajectory. Both sessions took about 40 min each. They were audio recorded and later transcribed verbatim and content analysis done and themes generated to enrich quantitative data. Finally, the transcripts were returned to the social workers and 7 patients (5 of them had died) who were in agreement that the information reflected their expressions.

### Data management and analysis

Quantitative data was coded, edited and cleaned to check for any errors and entered in Statistical Package for Social Scientists (SPSS) version 20 and presented in tables. Descriptive statistics and crosstabulations was done to analyze how cervical cancer patients differed in their financial challenges by their socio-demographic and clinical characteristics.

For qualitative data, the responses were transcribed verbatim; the content was then analyzed by searching for emerging themes on the financial challenges faced by cervical cancer patients. Qualitative data was presented in textual form with verbatim reports alongside quantitative data for illustrations.

## Results

### Socio-Demographic and Clinical Characteristics of Respondents

A total of 334 cervical cancer patients participated in the study. Those aged between 36 and 46 years were 114 (34%), with 93 (28%) aged between 18 and 35 years, 52 (16%) aged between 47 and 57, while 75 (22%) were aged 58 years and above. One hundred and seventy-eight (53%) of the survey respondents were widowed, 104 (31%) were married, 31 (10%) divorced or separated while only 21 (6%) were single. One seventy-nine (54%) of the respondents had primary level of education, 62 (18%) had secondary education and 43 (13%) had no education at all while only 50 (15%) had tertiary education. One twenty-nine (54%) of the respondents were at cancer stage IV, 63 (19%) were at stage III, 52 (15%) at stage I and 40 (12%) were at stage II. Two hundred and twenty-two (67%) of the respondents had not undertaken routine screening for cervical cancer previously, while only 33% had been routinely screened.

Two hundred and seventeen (65%) respondents were diagnosed of cervical cancer in less than one-year period prior to the study while only 31 (9%) were diagnosed in more than one year before the study. The rest of the patients did not know when they were diagnosed. Two hundred and nine (63%) of the respondents were Human Immunodeficiency Virus (HIV) Positive while only 73 (22%) were negative. The rest of the patients did not know their HIV status.

One fifty-nine (48%) respondents engaged in small scale farming whilst 145 (43%) were engaged in small scale business. Only 30 (9%) were formally employed.

One hundred and five (31%) of the patients received blood transfusion and pain killers and 74 (22%) received pain killers only. Most patients reported being put on antibiotics, haematemics for blood boosting and pain killing drugs such as aspirin, dichlophenac, brufen, intramusculine morphine and paracetamol. Thirty-two (10%) of the respondents received chemotherapy, haematemics 40 (12%), while only 11 (3%) of the respondents received radiotherapy and chemotherapy. Thirty (9%) of the patients received surgery while 42 (13%) were treated by LEEP.

### Financial challenges of cervical cancer patients

The key financial challenges from the study were costs of medication 291 (87%), cost of travel 281 (84%) and cost of diagnostic tests 250 (75%). Other costs incurred by the patients were cost of cloths and wigs 91 (27%), and cost of home and child care 80 (24%). Table 1 shows financial challenges experienced by patients.

**Table 1:** Financial challenges by cervical cancer patients

Variable	Yes	No	Total
Cost of treatment	228 (68%)	106 (32%)	334 (100%)
Cost of medication	291 (87%)	43 (13%)	334 (100%)
Cost of home or child care	80 (24%)	254 (76%)	334 (100%)
Cost of clothes and wigs	91 (27%)	243 (73%)	334 (100%)
Cost of diagnosis	250 (75%)	84 (25%)	334 (100%)
Travel costs	281 (84%)	53 (16%)	334 (100%)
Cost of nutritional supplements/	217 (65%)	117 (35%)	334 (100%)

Further in-depth interviews with patients revealed the theme of hygienic needs. Most patients incurred a lot of money in maintaining their personal hygiene. They stated that keeping clean as a woman was a great challenge that they were facing. Bleeding, discharge, and pus from their private parts really irritated them; in order to keep somehow clean, they had to use pads, swabs and at times medication or any substance that could counter foul smell that emanated from their private parts.

Key informant interviews with two medical social workers on the status of the facility as regards management of cervical cancer patients revealed the theme of inadequate resources. They stated that the facility had inadequate resources for managing cancer*.* For instance, the radiotherapy machine was not operational since it had broken down sometime back, there was lack of specialized diagnostic machines and oncologists, finance allocated for palliative care and oncology unit was insufficient for supplies required. Due to these challenges the patients were forced to seek some diagnostic tests in the private facilities which were very expensive for them. The care providers were forced to refer them for treatment to other facilities far away such as KNH or Mulago Hospital in Uganda hence escalating costs for them. Faced with such situations, most of the patients just gave up treatment and went back home.

### Health insurance cover

Three hundred and four (91%) of the cervical cancer patients admitted and referred to JOOTRH did not have adequate insurance cover and only 30 (9%) had National Hospital Insurance Fund cover which catered for only bed component of inpatient costs.This in most cases did not include medication such as chemotherapy, painkiller (morphine) which patients were supposed to buy from their out of pocket expenses.

### Financial assistance received by patients

Results showed that no patient received any assistance from well-wishers. Only a few 43 (13%) received assistance from charity organizations such as (Tumaini la maisha health services, and Kenya Medical Research Institution- Center for Disease Control), 91 (27%) received little assistance from friends, while 31 (9%) received little assistance from colleagues. Some patients received a little assistance from relatives 32 (10%) and church 32 (10%). Table 2 illustrates the results.

**Table 2:** Financial Assistance

Sources of financial assistance	None n (%)	Little n (%)	Moderate n (%)	Substantial n (%)
Friends	243 (73%)	91 (27%)	0 (%)	0 (%)
Colleagues	303 (91%)	31 (9%)	0 (%)	0 (%)
Relatives	84 (25%)	218 (65%)	32 (10%)	0 (%)
Well-wishers	334 (100%)	0 (%)	0 (%)	0 (%)
Charity Organizations	270 (81%)	43 (13%)	0 (%)	21 (6%)
Church	136 (40%)	156 (47%)	32 (10%)	10 (3%)

Key, *None*: Did not receive any financial assistance, *Little*: Received about 10% of total cost, *Moderate*: Received about 30% of total cost, *Substantial*: Received about 90% and above of total cost, *well-wisher*: A person who is not a friend, colleague or a church-mate to a cervical cancer patient, and not a member or affiliated to any charity organization but offers to give financial assistance to a patient.

Further interview with a medical social worker revealed the theme of lack of reliable source of financial assistance. He stated that patients did not receive substantial assistance, from the hospital apart from waiver that very few needy cases could get to help offset their hospital bill. There was no charity organization that was targeting cervical cancer patients, the few who benefited from charity organizations ware actually HIV/AIDS clients on care whose cases were considered as complications of HIV. He expressed a feeling that cervical cancer patients needed similar concerted effort from government and charity organizations in order for them to manage their condition.

Further inquiry revealed a theme of high cost of treatment. This meant that most of the patients were not able to cater for their treatment. He explained that most of the patients were poor and could not afford cancer treatment, most times they were referred for further treatment to KNH or Uganda or at times told to buy drugs for chemotherapy but they could not afford. Some of them were abandoned in the hospital and stayed until they were given waiver or died. Others went back home when they could not meet costs of chemotherapy.

In- depth interviews with patients further showed that most of the patients depended on their relatives, church members and their children for financial assistance most of whom did not have any formal employment or steady source of income. Hence the assistance was too little for the need they had.

### Financial challenges by participants sociodemographic and clinical characteristics

A larger proportion of Cervical cancer stage IV respondents experienced financial challenges as compared to other lower stages. For instance, cost of treatment was 146 (64%), cost of medication 168 (58%) and cost of diagnosis 158 (63%). Similarly, large proportion of widows experienced financial challenges as compared to other marital status. For instance, cost of treatment was 134 (59%), cost of medication 156 (54%) and cost of travel 167 (59%). HIV positive respondents bore disproportionate burden of financial challenges as seen in cost of treatment 135 (59%), and cost of travel 177 (63%). Respondents who had formal employment experienced lowest proportion of financial challenges as seen in cost of treatment 20 (9%), and cost of diagnosis 20 (8%). A larger proportion of Participants who attained primary level of education reported experiencing financial challenges compared to other levels of education. For instance, cost of treatment 136 (60%) and cost of diagnosis 136 (54%). Similarly, larger proportion of respondents who were treated by blood transfusion and pain killers reported financial challenges than the rest of the treatment modalities. Ninety-four (41%) reported challenges with cost of treatment while 94 (38%) reported challenges with cost of diagnosis. While a smaller proportion of insured participants experienced financial challenges compared to the non-insured as illustrated in Table 3.

**Table 3:** Financial challenges by participants sociodemographic and clinical characteristics

Characteristics	Cost of treatment (%)	Cost of Medication (%)	Cost of home and childcare (%)	Cost of clothes and wigs (%)	Cost of diagnosis (%)	Cost of travel (%)	Cost of Nutritional supplements (%)	N (%)
Cancer stage								
I	0	41 (14)	0	0	10 (4)	42 (15)	30 (14)	52 (16)
II	30 (13)	30 (10)	30 (38)	30 (33)	30 (12)	30 (11)	20 (9)	40 (12)
III	52 (23)	52 (18)	10 (12)	10 (11)	52 (21)	52 (18)	42 (19)	63 (19)
IV	146 (64)	168 (58)	40 (50)	51 (56)	158 (63)	157 (56)	125 (58)	179 (54)
Marital status								
Married	73 (32)	94 (32)	20 (5)	31 (34)	84 (34)	83 (30)	73 (34)	104 (31)
Divorced	10 (4)	20 (7)	10 (12)	10 (11)	10 (4)	20 (7)	20 (9)	31 (9)
Widowed	134 (59)	156 (54)	50 (63)	50 (55)	135 (54)	167 (59)	114 (52)	178 (53)
Single	11 (5)	21 (7)	0	0	21 (8)	11 (4)	10 (5)	21 (7)
HIV Status								
Positive	135 (59)	177 (61)	50 (63)	50 (55)	136 (54)	177 (63)	124 (57)	209 (63)
Negative	52 (23)	73 (25)	20 (25)	20 (22)	73 (29)	63 (22)	62 (29)	73 (22)
Don’t now	41 (18)	41 (14)	10 (12)	21 (23)	41 (17)	41 (15)	31 (14)	52 (15)
Income								
SC Farming	105 (46)	127 (44)	20 (25)	31 (34)	117 (47)	127 (45)	85 (39)	159 (48)
SC Business	103 (45)	134 (46)	40 (50)	40 (44)	113 (45)	124 (44)	102 (47)	145 (43)
Employed	20 (9)	30 (10)	20 (25)	20 (22)	20 (8)	30 (11)	30 (14)	30 (9)
Education								
None	10 (4)	32 (11)	10 (13)	10 (11)	32 (13)	43 (16)	10 (5)	43 (13)
Primary	136 (60)	147 (51)	30 (37)	41 (45)	136 (54)	147 (52)	95 (44)	179 (53)
Secondary	52 (23)	62 (21)	20 (25)	20 (22)	52 (21)	51 (18)	62 (28)	62 (19)
Tertiary	30 (13)	50 (17)	20 (25)	20 (22)	30 (12)	40 (14)	50 (23)	50 (15)
Treatment								
Chemo	32 (14)	32 (11)	10 (11)	10 (11)	32 (13)	32 (11)	32 (15)	32 (10)
Chemo+rad	11 (5)	11 (4)	0	0	11 (4)	11 (4)	11 (5)	11 (3)
Surgery	20 (9)	20 (7)	20 (25)	20 (22)	20 (8)	20 (7)	10 (5)	30 (9)
Bld tran+Pan k	94 (41)	94 (32)	0	11 (12)	94 (38)	94 (34)	63 (29)	105 (31)
Pain killers	31 (14)	63 (21)	20 (25)	20 (22)	63 (25)	42 (15)	41 (19)	74 (22)
Haematemics	40 (17)	40 (14)	30 (37)	30 (33)	30 (12)	40 (14)	40 (18)	40 (12)
LEEP	0	31 (10)	0	0	0	42 (15)	20 (9)	42 (13)
Insurance								
Yes	30 (13)	30 (10)	20 (25)	20 (22)	30 (12)	30 (10)	30 (14)	30 (9)
No	198 (87)	261 (90)	60 (75)	71 (78)	220 (88)	251 (90)	187 (86)	304 (91)
Total	228 (100)	291 (100)	80 (100)	91 (100)	250 (100)	281 (100)	217 (100)	334 (100)

KEY

*Chemo*: chemotherapy, *Bld tran + Pan k*: Blood transfusion and pain killers, *Chemo + rad*: chemotherapy and radiotherapy

*SC Farming*: small scale farming, *SC Business*: small scale business

## Discussion

Results from this study revealed several financial challenges that cervical cancer patients experienced. Majority of the patients reported engaging in small scale farming and business with only a few in formal employment, this implied low income status of the patients**.** Moreover, the patients did not have adequate medical cover to relieve their financial burden. As a result, the patients largely needed financial support to enable them meet costs of care. This agrees with.

Gitonga [20] who established that the total cost of breast cancer treatment at Kenyatta National Hospital (KNH) in Kenya is well above the country’s average wage level and subsidization is required. The patients experienced challenges with their treatment in terms of cost and access in that the treatment was unaffordable and inaccessible. Most patients could not go to KNH or Mulago hospital in Uganda where they were referred for treatment due to financial challenges. Cervical cancer patients also experienced financial challenges in acquisition of nutritional supplements which they needed to boost their health status. Most patients incurred cost of medication, travel and diagnostic tests. In contrast, fewer patients incurred cost of cloths and wigs, and cost of home and child care. This may be explained by the existence of strong extended family ties from which most patients benefited by receiving free home and child care assistance. The fewer number of patients reporting incurring costs of clothes and wigs can be explained by the fact that most patients in this study were not on treatment modalities that interfered with their body, such as hair loss or increase in weight. Most patients despite being in stage III& IV of the disease, were only treated with blood transfusion, haematemics and pain killers. They may not have required clothes and wigs which are expenses associated with those who receive chemotherapy and radiotherapy treatments. This concurs with Masika et al. [16] who also revealed that Tanzanian cancer patients incurred a lot of costs such as medication, travel, food, water, home and child care among others. Despite the fact that most patients were poor and could hardly bear the costs, the patients expressed a need for financial support to cater for these costs. Similarly, increasing demand for financial assistance and financial challenges among cancer patients have been reported in Ireland [3]. There is growing awareness that cancer can have major financial impact on newly diagnosed patients, those living with the disease, and their families [4]. This study finding differs with Aniebue et al. [22], report on ethical, socioeconomic, and cultural considerations in gynecologic cancer care in South Africa, which revealed that cancer treatment was free for patients who earn less than an established minimum income. The same report also revealed that in India surgery, radiotherapy, and chemotherapy treatments were partially or totally subsidized by government depending on a set minimum income level. Financial needs among cancer patients have also been reported by Sepulveda et al. [17] in their study conducted in a couple of African countries (Botswana, Ethiopia, Tanzania, Uganda and Zimbabwe) on quality of care at the end of life among cancer patients. Similarly, Murray et al. [23] and Masika et al. [16] also revealed need for financial support among cancer patients in Kenya and Tanzania respectively. Other international studies have also shown increasing need for financial assistance among cancer patients [4–6].

From the qualitative interviews it was evident that most patients were poor and could not afford treatment, this made some of them to abandon treatment and go back home while others just stayed in the ward with minimal treatment till they succumbed. This agrees with Mostert et al. [18] who studied contributors to abandonment of childhood cancer treatment in Kenya and established financial burden of cancer treatment and lack of insurances as one of the important predictors to abandonment of treatment. Similarly, in the US more than 2 million cancer survivors did not get one or more needed medical services because of financial concerns this was mainly seen among the Hispanic and black survivors [15]. Due to unique nature and symptoms of cervical cancer which includes constant bleeding and discharge from vagina, the patients also faced challenges with maintaining personal hygiene which led to increased financial needs. This concurs with Timmons et al. [7] who noted that patients incurred a wide range of additional cancer related medical and non- medical expenses in Ireland revealing the multidimensional nature of the financial and economic burden cancer imposes on patients.

This study also exposed resource-based challenges such as lack of radiotherapy and diagnostic machines, lack of oncologist and inadequate supplies for palliative care. This concurs with Louise et al. [19] who also reported lack of decentralized diagnostic and treatment facilities and a lack of better cancer policy as factors hindering access to cancer treatment in Kenya.

Further analysis of financial challenges by participants characteristics showed that patients who were at cancer stage IV, widows, HIV positive patients and those with lower education bore disproportionate burden of financial challenges.

This study was limited to cervical cancer patients seeking care at JOOTRH, this would mean that patients who were sick at home and could not access care at the hospital were left out. Such patients may be experiencing worse financial challenges but given that JOOTRH is a public hospital which is generally not so costly and has a waiver system for extremely needy patients, the patient population was deemed representative. Similarly, patients may have exaggerated their financial challenges with the hope that they would receive some assistance but this could be corroborated by the actual observable experiences they faced.

Based on the study findings, there exists numerous financial challenges that cervical cancer patients experience in the regional facilities, these range from treatment, diagnosis, travel and personal maintenance challenges.

## Conclusions

This study reveals that cervical cancer patients experience numerous financial challenges and there is a need for the health care system to develop mechanisms for supporting them especially patients in cancer stage IV, widows, HIV positive patients and those with low education as they bore the largest brunt of financial challenges. The government should increase budgetary allocation for cancer management and explore modalities of subsidizing cervical cancer treatment to enable most patients to afford. The wider society also need to be sensitized to offer support to members of the society suffering from life threatening diseases such as cervical cancer among them. Patients should be screened for financial challenges and its impact on their quality of life assessed. Finally, more studies should be done focusing on actual costs incurred by cervical cancer patients.

## Additional files

Additional file 1: Questionnaire for Cervical Cancer Patients. Perception of Cervical Cancer Patients on their Palliative Care Needs at Jaramogi Oginga Odinga Teaching and Referral Hospital in Western Kenya. Socio- Demographics and health history of cervical cancer patients that is presented in this paper. Financial challenges of cervical cancer patients that is presented in this paper. Health insurance cover status of cervical cancer patients that is presented in this paper. Sources of financial assistance for cervical cancer patients that is presented in this paper. Other data not presented in this paper: Patient Care and informational needs. Spiritual needs of cervical cancer patients. (DOCX 36 kb)

Additional file 2: Interview Guide for Healthcare Providers. Perception of Cervical Cancer Patients on their Palliative Care Needs at Jaramogi Oginga Odinga Teaching and Referral Hospital in Western Kenya. Physical and material needs of cervical cancer patients -Entails financial challenges, financial assistance and health insurance cover that is presented in this paper. Other data not presented in this paper: Biographical information of health care providers. Psychosocial needs of cervical cancer patients. Informational needs of cervical cancer patients. (DOCX 16 kb)

Additional file 3: In-Depth Interview Guide for Cervical Cancer Patients. Perception of Cervical Cancer Patients on their Palliative Care Needs at Jaramogi Oginga Odinga Teaching and Referral Hospital in Western Kenya. Physical and material needs of cervical cancer patients- Entails financial challenges, financial assistance and health insurance cover that is presented in this paper. Other data not presented in this paper: Psychosocial needs of cervical cancer patients. Informational needs of cervical cancer patients. Additional concerns of cervical cancer patients. (DOCX 17 kb)

## Abbreviations

GOPC: Gynecological out-patient clinic
HIV/AIDS: Human Immunodeficiency Virus/ Acquired Immune Deficiency Syndrome
JOOTRH: Jaramogi Oginga Odinga Teaching and Referral Hospital
KNH: Kenyatta National Hospital
LEEP: Loop Electrosurgical Excision Procedure
SPSS: Statistical Package for Social Scientists
