# Supplementary material for: Perception of Cervical Cancer Patients on their Financial Challenges in Western Kenya
Source: BMC Health Serv Res. 2018 Apr 10;18:261. doi: 10.1186/s12913-018-3073-2 (PMC5891984; doi:10.1186/s12913-018-3073-2)
Supplement: Supplementary file 1 — Questionnaire for Cervical Cancer Patients. Perception of Cervical Cancer Patients on their Palliative Care Needs at Jaramogi Oginga Odinga Teaching and Referral Hospital in Western Kenya. Socio- Demographics and health history of cervical cancer patients that is presented in this paper. Financial challenges of cervical cancer patients that is presented in this paper. Health insurance cover status of cervical cancer patients that is presented in this paper. Sources of financial assistance for cervical cancer patients that is presented in this paper. Other data not presented in this paper: Patient Care and informational needs. Spiritual needs of cervical cancer patients. (DOCX 36 kb) [file 12913_2018_3073_MOESM1_ESM.docx]

**ADDITIONAL FILE 1**

**QUESTIONNAIRE FOR CERVICAL CANCER PATIENTS**

**Perception of Cervical Cancer Patients on their Palliative Care Needs at Jaramogi Oginga Odinga Teaching and Referral Hospital in Western Kenya.**

Date…………………………………………………………………………………

Name of research assistant………………………………………………………….

Patient code…………………………………………………………………………

To the respondents,

All the information gathered herein, shall be kept confident and the report will not include the name of the informant. You are welcome to participate.

PART 1

SOCIO- DEMOGRAPHICS AND HEALTH HISTORY

1. Age

18-35(yrs.) [ ] 36 -46(yrs.) [ ]

47-57(yrs.) [ ] 58 and Above [ ]

1. Marital status

Married [ ] Divorced/Separated [ ]

Widowed [ ] Never married/single [ ]

1. Highest level of education attained

None [ ] Primary [ ]

Secondary [ ] Tertiary [ ]

No religion [ ]

1. What are your family’s main sources of income?

Small scale farming [ ] Small scale business [ ]

Large scale farming [ ] Large scale business [ ]

Formal employment [ ]

1. Does your family own land?

Not at all [ ] Less than an acre [ ]

Less than 2 acres [ ] More than 3 acres [ ]

1. Indicate sources of your drinking water

Well [ ] Stream [ ]

Bore-hole [ ] River [ ]

Tap [ ]

1. What type of energy do you use in your home for cooking?

Electricity [ ] Gas [ ]

Charcoal [ ] Solar [ ]

Kerosene [ ] Firewood [ ]

1. What type of human waste facility do you use?

Toilet [ ] Latrine [ ]

Nearby bush [ ] VIP [ ]

1. Cervical cancer stage

Stage I [ ] Stage II [ ]

Stage III [ ] Stage IV [ ]

Don’t know [ ]

1. Have you been screened for cervical cancer?

Yes [ ] No [ ]

1. When were you diagnosed of cancer?

Less than a year ago [ ] More than a year ago [ ]

1. Could you tell me your HIV status?

Positive [ ] Negative [ ] Don’t know [ ]

1. Treatment received in the last month

Chemotherapy [ ] Radiotherapy [ ]

Surgery [ ] Immunotherapy [ ]

LEEP [ ] Hormone treatment [ ]

Bone marrow treatment [ ] Other (Specify) ………………

**PART 2**

1. **Financial challenges of cervical cancer patients**

What are some of the costs you have covered during your illness period?

|  | Yes | No |
| --- | --- | --- |
| I have had to cover Cost of treatment |  |  |
| I have had to cover Cost of diagnosis |  |  |
| I have had to cover Cost of medication |  |  |
| I have had to cover Cost of nutritional supplements |  |  |
| I have had to cover Cost of home or child care |  |  |
| I have had to cover Cost of clothes and wigs |  |  |
| I have had to cover Travel costs |  |  |

According to your experience how would you describe your financial needs?

| No-Need  [ ] | Little Need  [ ] | Don’t know  [ ] | Moderate Need  [ ] | High need  [ ] |
| --- | --- | --- | --- | --- |

**Have you received any financial assistance from any of the following sources?**

|  | None | Little | Moderate | Substantial |
| --- | --- | --- | --- | --- |
| Friends |  |  |  |  |
| Colleagues |  |  |  |  |
| Relatives |  |  |  |  |
| Well-wishers |  |  |  |  |
| Charity Organizations |  |  |  |  |
| Church |  |  |  |  |

Key

None- No financial assistance

Little- About 10% of total cost

Moderate -  About 30% of total cost

Substantial- About 90% and above of total cost

**Do you have any health insurance cover?**

Yes [ ] No [ ]

If yes in (a) above does it cover your current illness?

Yes [ ] No [ ]

If yes in (b) above has it been adequate for your financial needs?

Yes [ ] No [ ]

b) **Patient Care and informational needs**

Patient support and care at the health facility-Below is a list of statements that describe patient/ provider relationship. Please tick one number per line to indicate your response as it applies to your experiences during the last one month

|  |  | Not at all | A Little bit | Somewhat | Quite abit | Very much |
| --- | --- | --- | --- | --- | --- | --- |
| 1 | Pharmacist advice on the proper use of my medicines |  |  |  |  |  |
| 2 | Advises me on the adverse (side) effects |  |  |  |  |  |
| 3 | Pharmacist help with the arrangements necessary to obtain my medicine |  |  |  |  |  |
| 4 | Hospital staff to convey a sense of hope to me and my family |  |  |  |  |  |

| 5 | Given the opportunity to talk to someone who understands and has been through a similar experience |  |  |  |  |  |
| --- | --- | --- | --- | --- | --- | --- |
| 6 | Given written information about the important aspects of my care |  |  |  |  |  |
| 7 | Given information (written, diagrams, drawings) about aspects of managing my illness and side effects at home |  |  |  |  |  |
| 8 | Given explanations of those tests for which I would like explanations |  |  |  |  |  |
| 9 | Adequately informed about the benefits and side effects of treatments before I choose to have them |  |  |  |  |  |
| 10 | Informed about my test results as soon as possible |  |  |  |  |  |
| 11 | Informed about cancer that is under control or diminishing (that is, remission) |  |  |  |  |  |
| 12 | Informed about things I can do to help myself get well |  |  |  |  |  |
| 13 | Informed about support groups in my area |  |  |  |  |  |
| 14 | Have access to professional counseling (e.g., psychologist, social worker, counselor,) if me/family/friends need it |  |  |  |  |  |
| 15 | Counseled on sexual matters |  |  |  |  |  |
| 16 | Treated with dignity |  |  |  |  |  |

According to your experience how would you describe Care and informational needs?

| No-Need  [ ] | Little Need  [ ] | Don’t know  [ ] | Moderate Need  [ ] | High need  [ ] |
| --- | --- | --- | --- | --- |

1. **Spiritual needs of cervical cancer patients**

The list that follows includes items which you may or may not experience, please consider how often you directly have this experience, and try to disregard whether you feel you should or should not have these experiences. A number of items use the word God. If this word is not a comfortable one for you, please substitute another idea which calls to mind the divine or holy for you.

|  | Not at all | A Little bit | Somewhat | Quite abit | Very much |
| --- | --- | --- | --- | --- | --- |
| I feel God’s presence |  |  |  |  |  |
| I find strength and comfort in my religion or spirituality |  |  |  |  |  |
| I feel deep inner peace or harmony |  |  |  |  |  |
| I ask for God’s help in the midst of daily activities |  |  |  |  |  |
| I feel God’s love for me directly |  |  |  |  |  |
| I feel God’s love for me through others |  |  |  |  |  |
| I desire to be closer to God or in union with Him |  |  |  |  |  |
| I need church members to visit me |  |  |  |  |  |
| I need spiritual group members to pray for me |  |  |  |  |  |
| I need to listen to gospel music |  |  |  |  |  |

According to your experience how would you describe your Spiritual needs?

| No-Need  [ ] | Little Need  [ ] | Don’t know  [ ] | Moderate Need  [ ] | High need  [ ] |
| --- | --- | --- | --- | --- |
