# Supplementary material for: Perception of Cervical Cancer Patients on their Financial Challenges in Western Kenya
Source: BMC Health Serv Res. 2018 Apr 10;18:261. doi: 10.1186/s12913-018-3073-2 (PMC5891984; doi:10.1186/s12913-018-3073-2)
Supplement: Supplementary file 2 — Interview Guide for Healthcare Providers. Perception of Cervical Cancer Patients on their Palliative Care Needs at Jaramogi Oginga Odinga Teaching and Referral Hospital in Western Kenya. Physical and material needs of cervical cancer patients -Entails financial challenges, financial assistance and health insurance cover that is presented in this paper. Other data not presented in this paper: Biographical information of health care providers. Psychosocial needs of cervical cancer patients. Informational needs of cervical cancer patients. (DOCX 16 kb) [file 12913_2018_3073_MOESM2_ESM.docx]

**ADDITIONAL FILE 2**

**INTERVIEW GUIDE FOR HEALTH CARE PROVIDERS**

**Title of the study-** (Perception of Cervical Cancer Patients on their Palliative Care Needs at Jaramogi Oginga Odinga Teaching and Referral Hospital in Western Kenya**)**

Date ……………………. Name of researcher………………………….

Provider code…………… Duration of practice in the unit……………...

*To the respondents- all the information gathered herein, shall be kept confident and the report will not include the name of the informant. You are welcome to participate.*

1. **Biographical information**
2. What is your area of specialization?
3. How long have you practiced palliative care?
4. What palliative care-oriented training have you attained?

Probes:

Therapeutic communication/ Counseling?

Cancer care needs assessment?

Psychosocial support?

Any other?

1. **Psychosocial needs of cervical cancer patients**
2. In your opinion what are some of the Psychosocial challenges that patients face under your care?

1. What are the mechanisms you have in place for providing cervical cancer patients and their families with professional counseling?

Probes:

Emotional counseling?

Sexual counseling to the patients and their spouses?

1. Do cervical cancer patients in your facility have support groups?

If yes, how do they access such groups?

1. What is your opinion on cervical cancer patients’ spiritual needs?

Do you in any way cater for cervical cancer patients’ spiritual needs?

If yes how?

1. How do you ensure that your patients are treated with dignity?
2. Going by your day to day interaction with the patients. Would you suggest some needs you think they have regarding psychosocial domain of wellbeing
3. **Physical and material needs of cervical cancer patients**
4. In your opinion what are some of the physical challenges that patients face under your care?
5. How do you deal with pain relief and symptom management among your

cervical cancer patients?

Probe: how available and affordable are the drugs that you use in this case?

1. What are some of the costs that your patients incur?
2. In cases where patients have insurance cover, is it always adequate for their financial needs?
3. How do you handle patients with serious financial challenges?

Probe: Are there any source of support you link them to?

1. Going by your day to day interaction with the patients. Would you suggest some needs you think these patients have regarding Physical and material domain of wellbeing?
2. **Informational needs of cervical cancer patients**
3. In your opinion do these patients need information regarding their disease treatment and care?

If yes**,** do you meet those needs? And how do you meet them

Probes:

- Information on managing illness side effects at home
- Benefits and side effects of treatment
- Nutritional information
- Availability of support groups

1. How prompt do you inform cervical cancer patients about their test results?

Probe:

Do you give explanations of those tests for which the patient would like explanations?

If yes how do you do this and who does it?

1. Going by your day to day interaction with the patients. Would you suggest some needs you think they have regarding Patient care and informational domain of well being
2. **General overview**

In your opinion are there any challenges you experience while providing the above services to your cervical cancer patients?

END

Thank you for finding time in your busy schedule to answer the above questions.
