# Supplementary material for: Perception of Cervical Cancer Patients on their Financial Challenges in Western Kenya
Source: BMC Health Serv Res. 2018 Apr 10;18:261. doi: 10.1186/s12913-018-3073-2 (PMC5891984; doi:10.1186/s12913-018-3073-2)
Supplement: Supplementary file 3 — In-Depth Interview Guide for Cervical Cancer Patients. Perception of Cervical Cancer Patients on their Palliative Care Needs at Jaramogi Oginga Odinga Teaching and Referral Hospital in Western Kenya. Physical and material needs of cervical cancer patients- Entails financial challenges, financial assistance and health insurance cover that is presented in this paper. Other data not presented in this paper: Psychosocial needs of cervical cancer patients. Informational needs of cervical cancer patients. Additional concerns of cervical cancer patients. (DOCX 17 kb) [file 12913_2018_3073_MOESM3_ESM.docx]

**ADDITIONAL FILE 3**

**IN-DEPTH INTERVIEW GUIDE FOR THE PATIENTS**

**Perception of Cervical Cancer Patients on their Palliative Care Needs at Jaramogi Oginga Odinga Teaching and Referral Hospital in Western Kenya.**

Theme 1-Psychosocial needs of cervical cancer patients

What psychosocial problems do you experience?

Probes –sadness, hopelessness, anxiety, worry, nervous,

loneliness, helplessness, inner loneliness (Gods absence in your

life)

What do you do to manage such experiences?

1. Are you attached to any support group?

How did you access them?

How helpful is it to you?

1. How supportive are your family members?
2. How do your family members and friends view and feel about

your condition?

1. What would you say regarding your sexual relationship with your spouse?
2. Do you feel you are treated like a person with dignity by your health care providers or just another case?

Theme 2- Physical and Material needs of cervical cancer patients

In your opinion what are some of the physical and material issues you face now.

Probes: Pain, lack of energy, nausea, lack of help with home and child

care, financial needs

1. What would you say about your pain relief and symptom management

Probe: how available and affordable are the drugs that you use in this

case?

1. What are some of the costs that you incur due to your condition?

Probes: treatment, travel, medication, home and or child care, nutritional

supplements, clothes and wigs

1. How do you meet these costs?

Probe: Are there any source of material /financial support you access?

1. From your experience would you suggest as some needs you have regarding Physical and material domain of wellbeing?

Theme 3 – Informational needs of cervical cancer patients

Do you receive any information or information material about aspects of care from your provider concerning the following topics?

Probes-

- 1. Available treatment options?
  2. Benefits and side effects of treatments?
  3. Management of side effects at home?
  4. Nutritional information?
  5. If you receive information, in what form is it provided, who gives it to you and is it sufficient for your needs?
  6. Do you receive your test results as soon as possible?
  7. Are you given the freedom to choose or contribute in choosing your treatment options?
  8. What would you say about this?
  9. Suggest the best way you feel can be used in providing for your informational needs?

Theme 4- Additional concerns

What are some of the difficulties you experience due to your health condition?
